# The relationship between knowledge about the pandemic and willingness to get vaccinated against SARS-CoV-2 in medical students in Poland: A cross-sectional survey

**DOI:** 10.3389/fpubh.2022.914462

**Published:** 2022-08-25

**Authors:** Aleksandra Jastrzẹbska, Gabrielle Saden, Brygida Knysz, Maciej Pondel, Agnieszka Siennicka

**Affiliations:** ^1^Department of Infectious Diseases, Liver Diseases and Acquired Immune Deficiencies, Wroclaw Medical University, Wroclaw, Poland; ^2^Business Intelligence in Management Department, Wroclaw University of Economics and Business, Wroclaw, Poland; ^3^Department of Physiology and Pathophysiology, Wroclaw Medical University, Wroclaw, Poland

**Keywords:** COVID-19, SARS-CoV-2, vaccination, education, medical students

## Abstract

The COVID-19 pandemic underlines the importance of targeting the groups with the highest risk of vaccine hesitancy, understanding their fears, and alleviating them. As the pandemic situation is very dynamic due to the appearance of new SARS-CoV-2 variants, concerns might also change over time. This is the first study to evaluate the vaccination rate and state of knowledge among medical students in Poland, comparing English and Polish divisions. We collected the data in 2 months. A total of 1,521 surveys were collected as follows: 273 students from the English division and 1,248 students from the Polish division answered the survey. The questionnaire was aimed at investigating students' awareness, knowledge, and apprehensions toward the COVID-19 pandemic and vaccines. The results were obtained for the following statements: good knowledge about ways of transmission is not statistically significant in determining if a student is vaccinated. Moreover, a year of study is not statistically significant in determining if a student knows all ways of COVID-19 transmission. Interestingly, the correlation between the statement “Keeping up to date with the upcoming vaccines is important for my role as a future health care worker” and being vaccinated against SARS-CoV-2 showed that 45.5% of unvaccinated students did not update their information about the vaccines and rated 1 out of 5 for this statement (*p* < 0.001). Even though the pandemic will not last forever, the obtained knowledge about the role of individual interests can be applied in many different life situations as this feature is statistically significant.

## Introduction

From the very beginning of the COVID-19 outbreak, the vaccination for SARS-CoV-2 was considered a possibility to terminate the pandemic. However, the development of the vaccine did not end the spread of the disease due to many factors, including vaccine reluctance (1), which delays obtaining herd immunity. Vaccination acceptance is geographically dependent. In Eastern Europe, the highest vaccination rate was reported in Montenegro (69%), while in Poland, the vaccination acceptance rate is estimated at 51% (2). A global survey of potential acceptance of the COVID-19 vaccine found Poland to be at the second-to-last place in a ranking performed in pre-vaccination times (3). The impact of vaccination hesitancy on different regions of the world might also be influenced by the levels of trust in governments. The evidence for this assumption might be an example of the higher vaccination acceptance in the Asia and Pacific regions in contrast to some parts of Africa (Cameroon, Senegal, and Liberia) (2). The reluctance to vaccinations is as old as their development and occurred first when smallpox vaccination was introduced by Edward Janner in 1796 (4), and it will always remain a disturbance also in possible future pandemics. Vaccine hesitancy is responsible for the recurrence of preventable diseases, for example, 53,219 cases of measles in Ukraine in 2018 (5). The COVID-19 pandemic underlines the importance of targeting the groups with the highest risk of vaccine hesitancy, understanding their fears, and alleviating them. Also, we should not perceive vaccinations as a solution to all our problems and neglect other nonspecific prevention, for example, social distancing, personal protective equipment (PPE), and hand hygiene (6, 7). As reported by Nguyen et al. (7), inadequate usage of personal protective equipment caused an increased risk of positive COVID-19 tests in frontline healthcare providers. The greatest risk was observed in nursing homes and inpatient settings.

As the pandemic situation is very dynamic due to the appearance of new SARS-CoV-2 variants, concerns might also change over time. The most common apprehensions related to vaccinations against COVID-19 seem to be safety, side effects, and doubts about their efficiency (8, 9). In a study performed by Sowa et al., it was found that people concerned about side effects were 82% less likely to be vaccinated. As reported by other researchers, vaccination acceptability may be influenced by many other factors such as religion, fitness index, national narcissism, and conspiracy theory index (10, 11). Additionally, a low level of education is a negative predictive factor of vaccination acceptance (12). Being a man is another feature that may be associated with increased vaccination compliance (1). To verify this data among students at medical universities in Poland, in both Polish divisions (PD) and English divisions (ED), we ran an anonymous survey.

The survey was aimed at investigating students' apprehensions toward vaccinations, concerns regarding the new SARS-CoV-2 variants, preferred educational tools for acquiring information about the pandemic, the presence of constantly updated COVID-19 curricula, and a clear system for monitoring infections at their universities, personal protective equipment providence, and compliance.

We hypothesized that (1) new variants of the virus might cause unwillingness to be vaccinated or receive booster shots, (2) students' awareness of the pandemic and willingness to be educated in this field is a strong positive predictor of being vaccinated, and (3) students from universities that provide suitable educational tools are better informed about the pandemic. We would like to highlight the appearance of the new SARS-CoV-2 (Omicron/B 1.1.529) shortly before launching the survey (21 December 2021), which might have disturbed the previous perspective on vaccination due to reports about the differences in this variant's S protein structure (13). Therefore, in our opinion, it is crucial to update information obtained during previous research on vaccination hesitancy among Polish students, which was done before the emergence of many SARS-CoV-2 variants (14). This is the first study to evaluate the vaccination rate and the state of knowledge among medical students in Poland, comparing English and Polish divisions.

## Materials and methods

### Data collection

We collected the data in 2 months from 21 December 2021 to 18 February 2022. Two questionnaires were created, i.e., a Polish version for students at PD and an English one for ED students. We designed the survey using Survio and it was distributed *via* universities' websites or it was sent directly to students' emails; we also used Facebook to reach students who are not interested in academic life and do not follow information shared by their university, in which case the link was shared in groups of years of departments such as medicine, dentistry, pharmacy, and health sciences at medical universities in Poland, both PD and ED if possible. Before sharing the survey with the respondents, we conducted a pilot survey among ten students (five from PD and five from ED) and consulted the marketing department at Wroclaw Medical University. All comments were incorporated.

Ethical approval was granted by the Bioethical Committee of Wroclaw Medical University before the study's initiation. On the front page, students were assured that the survey was voluntary and anonymous, that the research did not involve any potential threats, and that the refusal to continue participation might be given at any time of completing the survey.

### Participants

Universities that took part in our research were Wroclaw Medical University, Medical University of Warsaw, Medical University of Gdansk, Poznan University of Medical Sciences, Faculty of Medical Sciences in Katowice, Medical University of Bialystok, Medical University of Lublin, Medical University of Lodz, and others (for students from other universities, there was an option to indicate their university in an open question).

### Study design

We used an original questionnaire designed specifically for the needs of this study. The questionnaire was divided into sections about (1) demographic data (including gender, age, university, department, and the year of studies), (2) students' awareness about the pandemic and emergence of new variants, (3) vaccinations, (4) masks, (5) social distancing, and (6) hand hygiene.

Students' awareness of the pandemic was measured by answers to the following questions: multiple choice questions about the ways of transmission of SARS-CoV-2, yes/no questions regarding the information provided by the university about COVID-19 symptoms, consequences, prophylaxis, and perception of personal exposure hazard.

The part about vaccination started with the question, “Are you vaccinated against COVID-19?” Subsequently, the participants were asked about their major concerns before being vaccinated or preventing them from being vaccinated. The vaccinated individuals were asked about taking the booster shot, and the unvaccinated individuals with a booster shot were asked if the appearance of a new SARS-CoV-2 variant might cause the postponement of their decision. To assess the role of universities in education about the pandemic, students answered a question about whether their educational institution provided sufficient access to information about COVID-19 symptoms/consequences/prevention and whether there existed a clear system for monitoring infections and contacts at their university. Regarding the creation of the curricula adapted to students' demands, we asked for a preferred educational tool.

The parts of the survey about masks, social distancing, and hand hygiene focused on the students' compliance and frequency of using these prophylaxis methods and the university's supplies of personal protective equipment.

### Statistical analysis

Statistical analysis was performed using Microsoft Power BI for visualizations, SciPy stats Python library for chi-square calculation, and Python xgboost package with XGBClassifier algorithm and Scikit-learn library with DecisionTreeClassifier algorithm for feature importance identification. In our analyses, a *p*-value of <0.05 was considered statistically significant.

Our sample did not include all students at Polish Medical Universities; however, our group was representative and enabled us to run all the statistical analyses.

**Figure 1:**
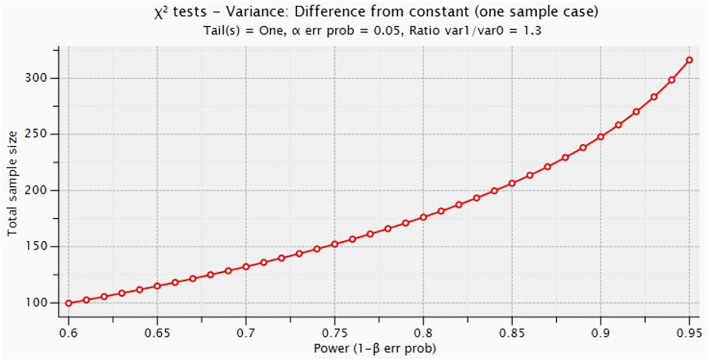
Simulations of the sample size test depending on the test power.

The analysis of the sample size was conducted based on one of the main primary aims in which it was assumed that at least 30% of the participants would declare to get vaccinated again despite the appearance of some new virus variants. The minimum number of the sample size calculation that is required to detect this difference while alpha = 5%, power = 90%, and the level of trust = 96% is 247 patients altogether. Additionally, the risk of the lack of the correct completion of the survey was assumed at the 10% level. The final sample size test equals 272 participants, which corresponds to the number of ED participants. The analysis of the sample size test was conducted using the G^*^Power program. Additionally, we attach simulations of the sample size test depending on the test power (Figure 1).

## Results

### Study group

A total of 1,521 surveys were collected: 273 students from the ED (overall completion rate: 32.4%) and 1,248 students from the PD (overall completion rate: 50.4%) completed the survey. Details are shown in Table 1. Notably, 96% of Polish division students and 94% of English division students were vaccinated against COVID-19.

**Table 1:** Demographic characteristics.

	**Polish division**	**English division**
Total; *n*	1,248	273
Female; *n* (%)	893 (71.6%)	156 (57.1%)
Median age	21	24
**University (% of participants)**		
Wroclaw Medical University	37.4%	39.2%
Medical University of Warsaw	3.0%	13.9%
Medical University of Gdansk	25.4%	3.3%
Poznan University of Medical Sciences	9.9%	12.8%
Faculty of Medical Sciences in Katowice	9.1%	0.7%
Medical University of Białystok	2.5%	7.0%
Medical University of Lublin	3.2%	0.4%
Medical University of Łódz	2.4%	1.5%
Other	7.1%	21.2%
**Department (% of participants)**		
Medical	54.7%	96.3%
Dentistry	8.8%	3.7%
Health sciences	20.0%	0.0%
Pharmacy	16.4%	0.0%
**Year of studies (% of participants)**		
I	30.4%	25.6%
II	15.2%	16.8%
III	17.9%	11.0%
IV	14.7%	22.0%
V	15.5%	9.5%
VI	6.3%	15.0%

### Students' apprehensions toward vaccinations

**Figure 2:**
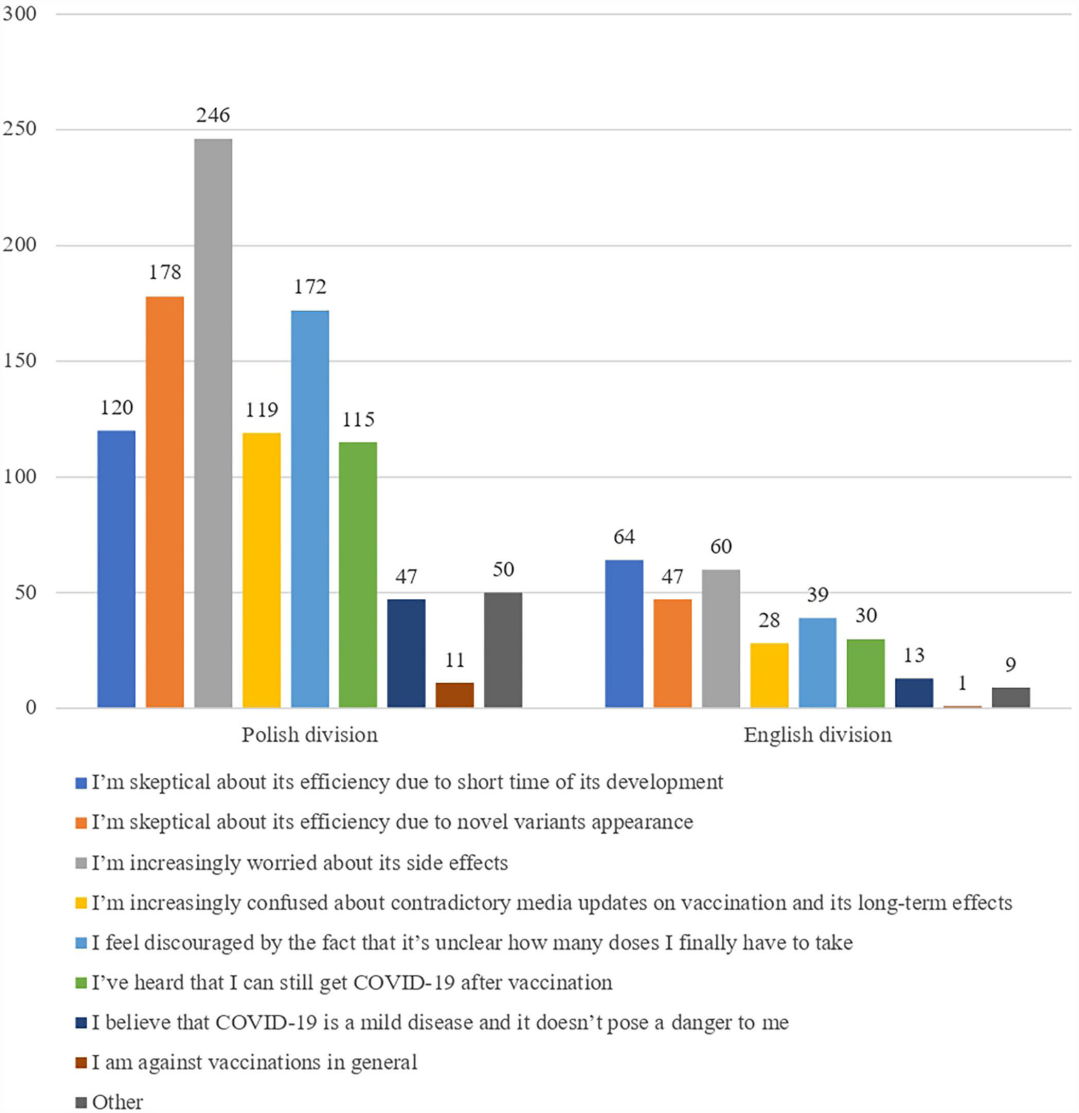
Students' apprehensions toward vaccination.

Students who were asked about their concerns before the vaccination gave the following answers (Figure 2).

One of the investigated issues was vaccination reluctance due to the appearance of new SARS-CoV-2 variants. Students who had not yet taken the third shot of the vaccine were asked if the appearance of the new variant of SARS-CoV-2 (B 1.1.529—“Omicron”) discouraged them from taking it. The negative answer to this question was given by 326 (69%) of students from both PD and ED, who had been vaccinated but did not take the third shot.

### Students' knowledge

**Figure 3:**
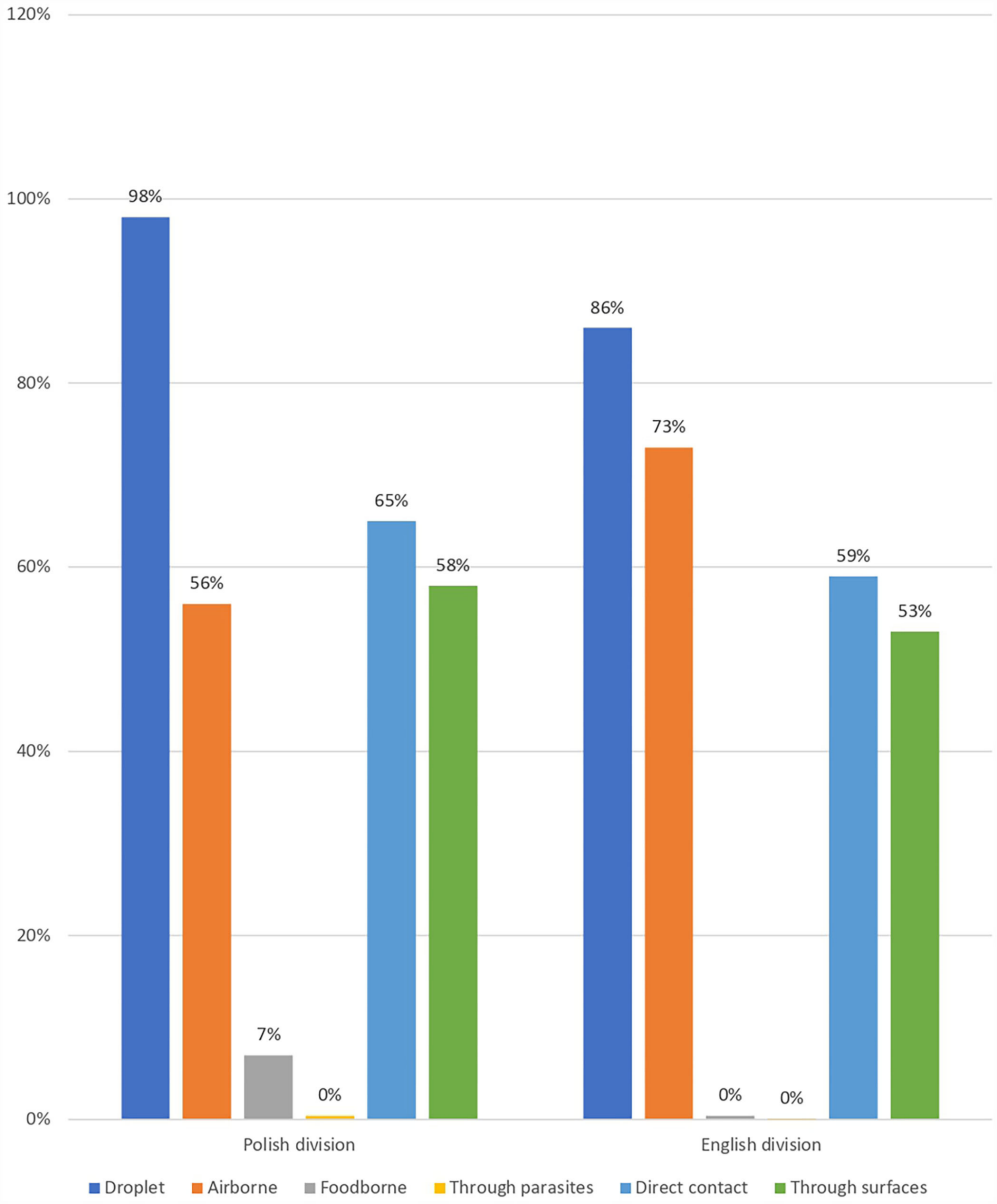
Students' knowledge about transmission ways of the SARS-CoV-2.

**Figure 4:**
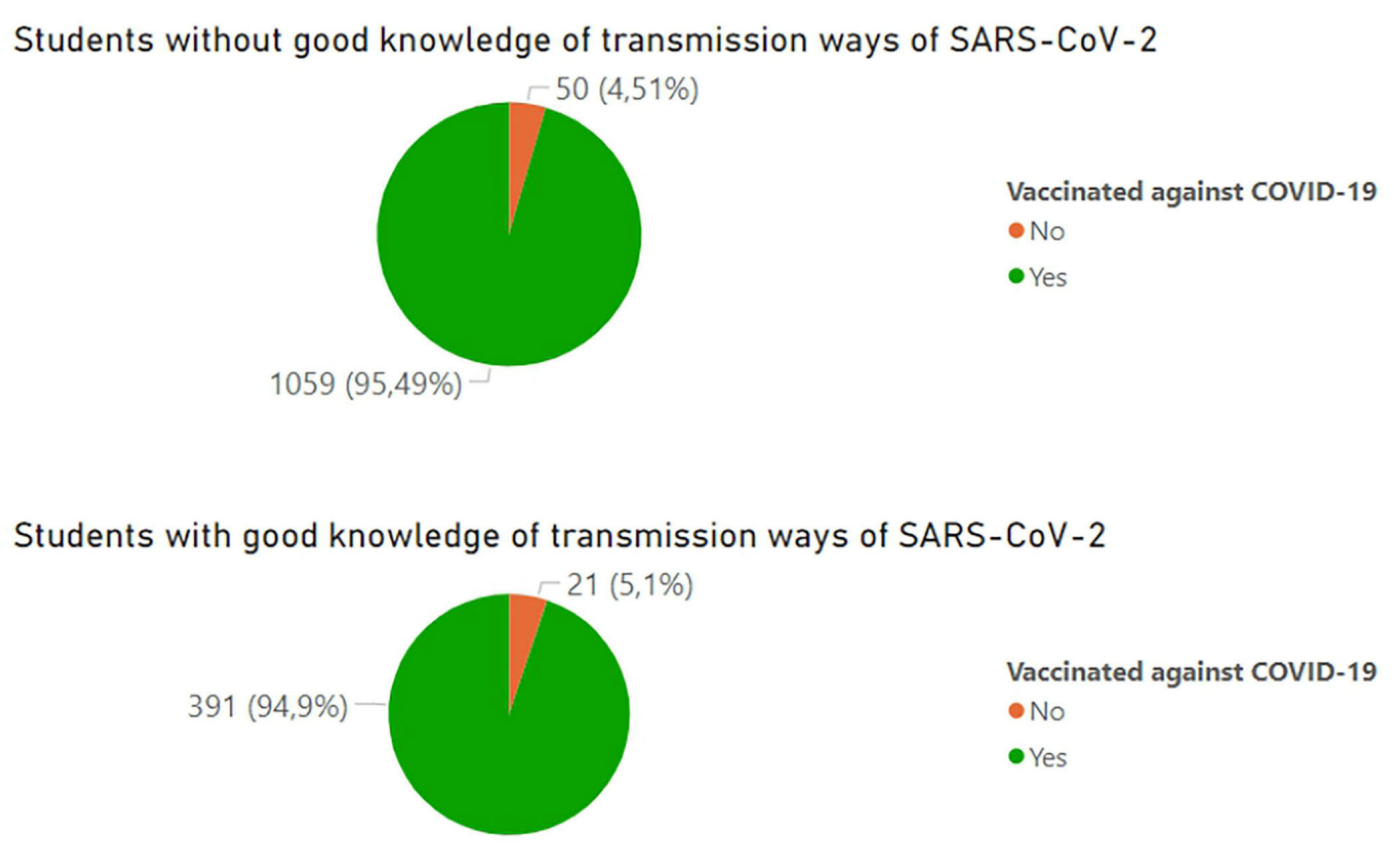
Odds of being vaccinated according to students' good knowledge of transmission ways of SARS-CoV-2.

**Figure 5:**
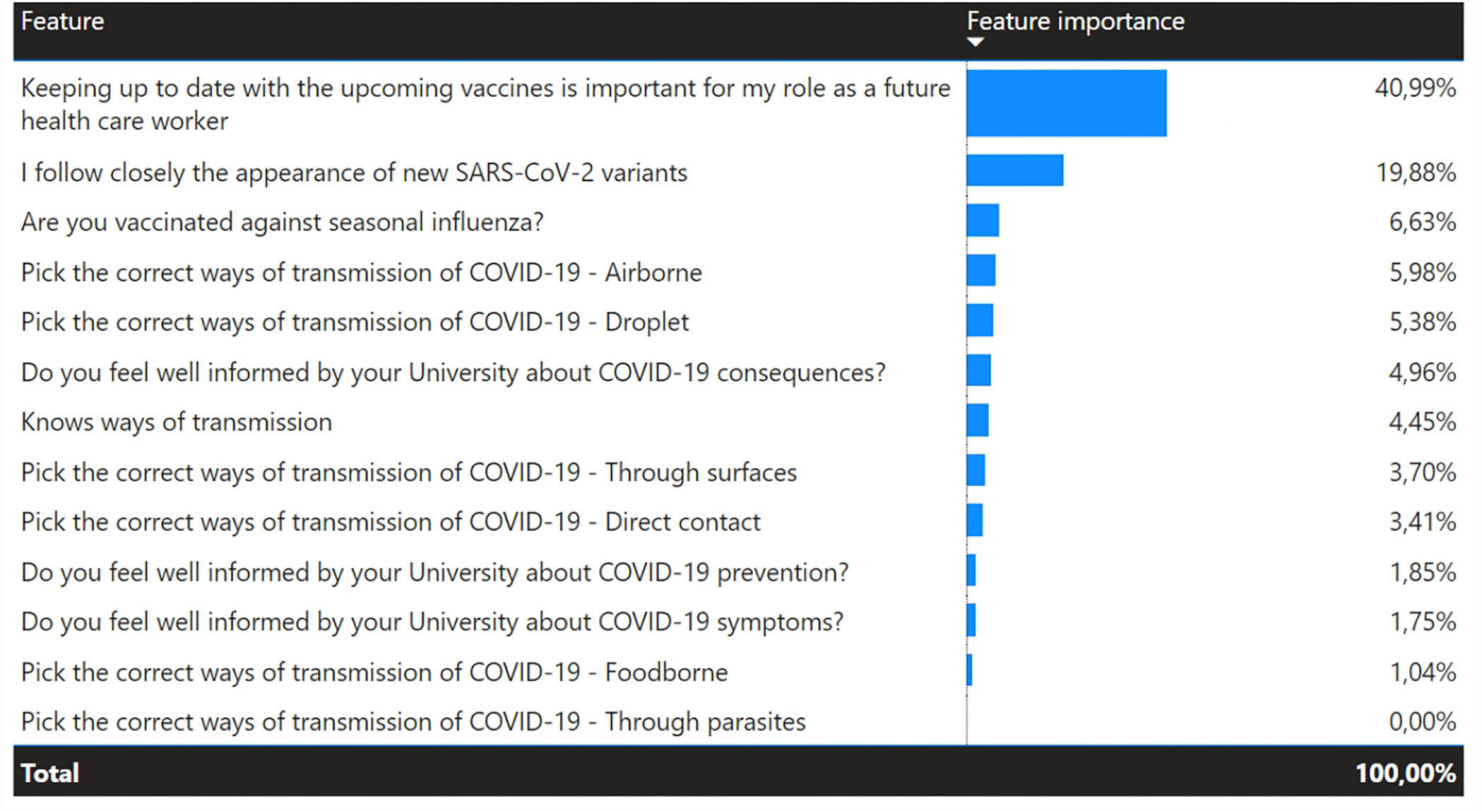
The most important features in vaccination prediction.

The question about the ways of SARS-CoV-2 transmission was a multiple-choice question with the following correct answers: droplet, airborne, direct contact, and through surfaces, and the distractor answers were as follows: foodborne and through parasites (Figure 3). Percentage value of students who pointed each way of transmission as relevant in terms of COVID-19 is presented in Figure 4. A fully correct answer to this question was given by 27.1% of all students. According to the chi-square test (chi-square-score = 3.49, *p* = 0.32), good knowledge of ways of transmission is not statistically significant in determining if a student is vaccinated. To determine the factors influencing the fact of vaccination, a decision tree was constructed using the Python programming language and Scikit-learn library. The DecisionTreeClassifier algorithm was used. The algorithm identified the most important features in vaccination prediction (Figure 5).

### The role of the university

The participating students were asked to answer the following question: Do you feel well-informed by your university about COVID-19 symptoms/consequences/prevention? At PD, 968 students (77.6%) felt well-informed by their university about COVID-19 symptoms, 799 (64.0%) about COVID-19 consequences, and 1,033 (82.8%) about COVID-19 prophylaxis. In ED, 206 students (75.5%) felt well-informed by their university about COVID-19 symptoms, 176 (64.5%) about COVID-19 consequences, and 215 (78.8%) about COVID-19 prevention. A year of studies is not statistically significant in determining if a student knows all ways of COVID-19 transmission (chi-square score = 7.997 and *p* = 0.16).

In PD, 95.7% of students were vaccinated against COVID-19 and in ED, 93.8% of students. Additionally, students were asked about vaccination against seasonal influenza with the following results: 42.1% were vaccinated in PD and 49.1% in ED. Being vaccinated against seasonal influenza is statistically significant in determining if a student is vaccinated against COVID-19 (chi-square score = 40.06 and *p* < 0.001).

In the group vaccinated against COVID-19, 9.7% of PD students and 14.1% of ED students were infected with SARS-CoV-2 and tested positive despite the vaccination. The average time between the last vaccination dose and the infection confirmed by a test was 141.62 days.

### Preferred educational tools

Focusing on students' preferred educational tools, multiple choice questions were asked with the following possibilities: to choose from short videos, lectures, tests before starting classes, articles, interviews with experts with the possibility of asking questions, and others. Both PD and ED students found short videos as the most suitable way of learning (PD students: 62.1%, ED students: 65.8%).

**Figure 6:**
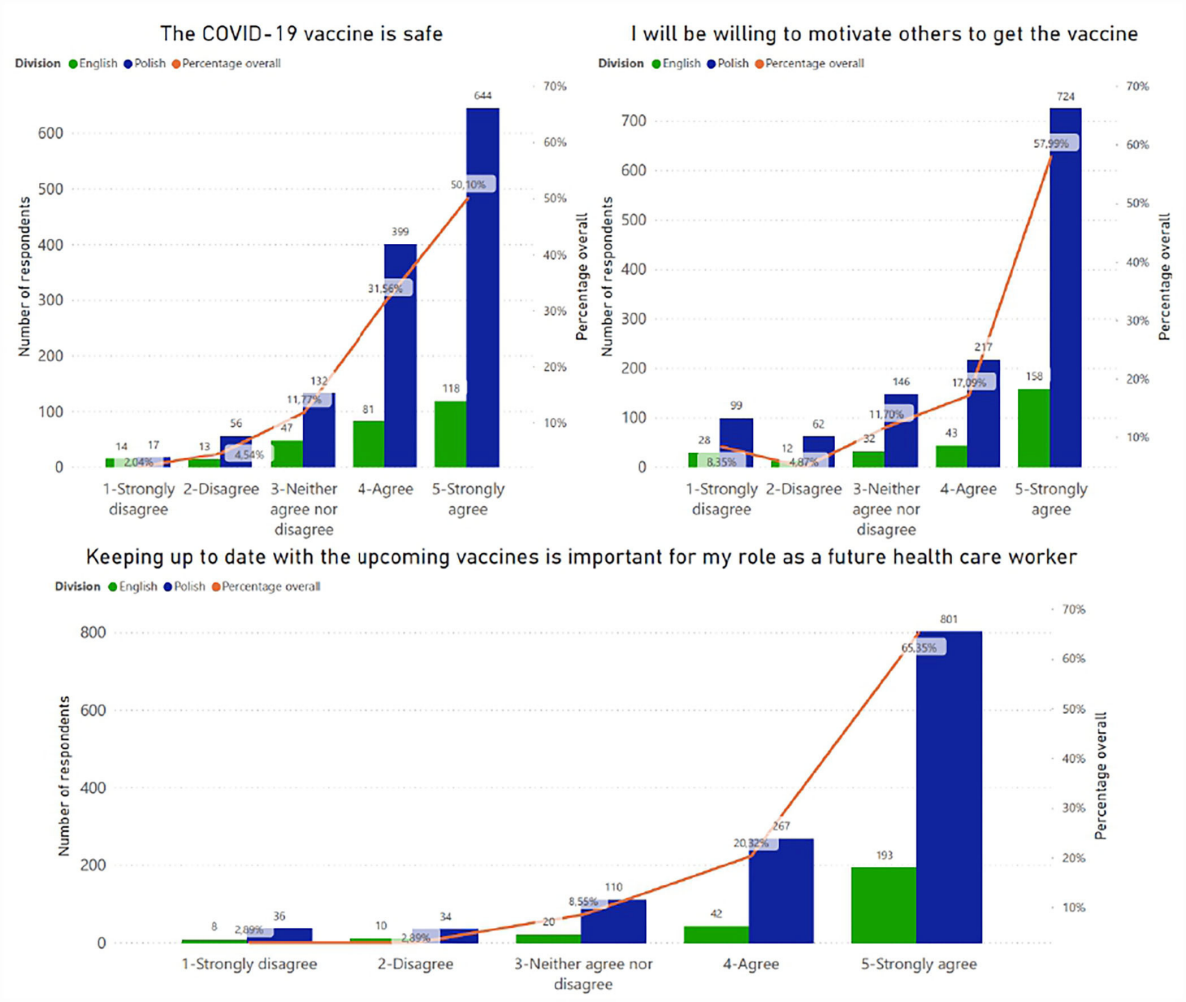
Students' opinions about different statements (measured by Likert scale).

**Figure 7:**
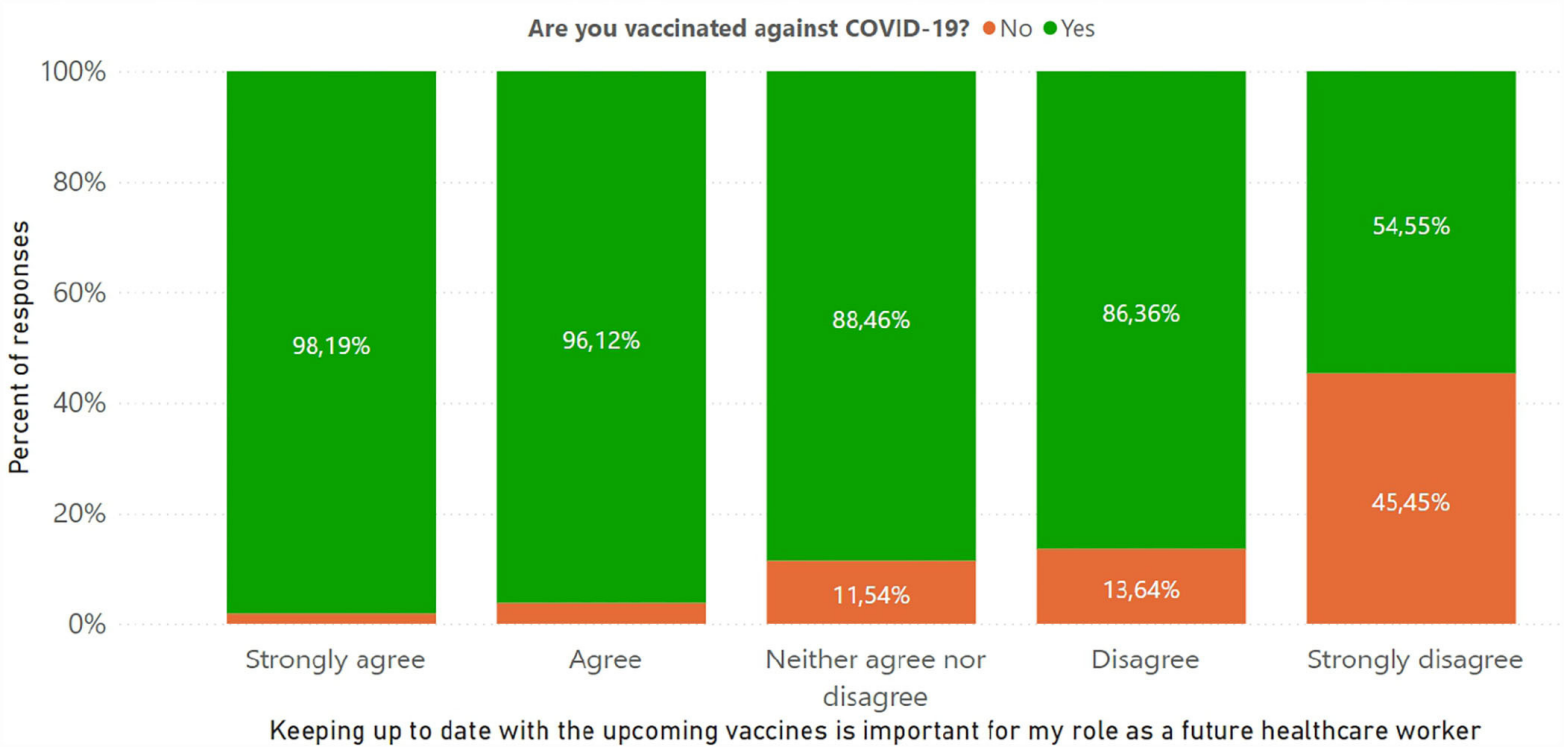
Correlation between vaccination status of students and the following statement “Keeping up to date with the upcoming vaccine is important for my role as a future health care worker.”

The part with questions with answers on a scale from 1 to 5 revealed the following results (Figure 6). Interestingly, the correlation between the statement “Keeping up to date with the upcoming vaccines is important for my role as a future health care worker” and being vaccinated against SARS-CoV-2 showed that 45.5% of unvaccinated students did not update their information about the vaccines and rated 1 out of 5 for this statement (*p* < 0.001) (Figure 7).

### Nonspecific prophylaxis

Regarding the questions about nonspecific prophylaxis, students were asked about wearing masks in all the places they were required. This question was answered positively by 79.1% of PD students and 81.3% of ED students. As the most common reason for not wearing masks, students claimed, “I have difficulties breathing while wearing a mask” (43.7% of PD students and 39.2% of ED students). Only 21.3% of PD students and 20.1% of ED students changed their masks more than once a day. The appearance of new SARS-CoV-2 variants, such as the Omicron, was not seen by 11.3% of PD and 14.7% of ED students as a threat and did not convince them to wear masks more often.

Questions about social distancing regulations revealed that 2.9% of PD and 2.6% of ED students never complied with the regulations; 22.3% of PD and 16.8% of ED students almost never complied with the regulations. As the most common cause of that students claimed “I find it impossible to constantly focus on social distancing in my everyday life.”

Details of the questions about hand hygiene are shown in Table 2.

**Table 2:** How often students wash and disinfect their hands at different moments of the university day.

	**Are you vaccinated against COVID-19?**	
	**Yes**	**No**
**How often do you wash your hands during your classes at the hospital?**		
Before examining a patient	21.80%	78.20%
After examining a patient	28.10%	71.90%
Before classes	27.20%	72.80%
After classes	36.50%	63.50%
While changing hospital department	12.20%	87.80%
Not at all	0.90%	99.10%
I only disinfect my hands	8.10%	91.90%
**How often do you disinfect**		
**your hands during classes at the hospital?**		
Before examining a patient	51.30%	48.70%
After examining a patient	50%	49.60%
Before classes	43.30%	56.70%
After classes	48%	52.00%
While changing hospital department	30%	70.00%
Not at all	0.50%	99.50%

## Discussion

We present the first study describing the state of knowledge among medical students in Poland, as well as their attitudes and fears regarding vaccination. A previous publication in the same country focused only on nursing students (15) and did not focus on the appearance of variants. Other studies in different countries based on anonymous surveys were conducted at a time when vaccines were not available to medical students.

### Awareness of SARS-CoV-2 virus and COVID-19 vaccine

As future healthcare workers, medical students have an important role to play in motivating the general public to get vaccinated. Previous studies (16) have shown that a broader knowledge of vaccination is correlated with a higher vaccination rate. Mayan et al. concluded that medical students with better knowledge were also more likely to receive the vaccine as opposed to those with worse knowledge, who were less inclined to receive the vaccine. Another study showed no association between the willingness to vaccinate and knowledge about the vaccine or the virus (17). We also found no correlation between vaccination status and knowledge of COVID-19. Moreover, students who answered positively to the statement “keeping up to date with the upcoming vaccines is important for my role as a future health care worker” were more likely to be vaccinated.

### Previous vaccination and booster shots

We found that the vast majority (95.3%) of students are vaccinated against COVID-19. It is a much better result than the results from a study conducted in Slovakia, where the coverage was 71.7% (18). A study of January 2022 in Spain got similar results, with 97.8% of students being vaccinated (19). They also found that the coverage against influenza was higher in students vaccinated against COVID-19 (35.5 vs. 14.3%). Among students who replied to our survey, slightly less than half of them are vaccinated against seasonal influenza. The coverage is also higher in students who are vaccinated against COVID-19 (45.17 vs. 7.04%).

Similar to the study conducted in Slovakia, being vaccinated against seasonal influenza is a positive predictor of vaccination against COVID-19. This may be explained by paying greater attention to public health recommendations, leading to greater compliance.

Among students who did not get a booster shot, the majority (69%) responded that the appearance of new variants did not discourage them from getting it. Our survey had the particularity to be launched just a few days after the appearance of the Omicron. This variant had the singularity to have more than 50 mutations, of which the virus' spike protein had 26–35 amino acids that were different from the original SARS-CoV-2 virus or the Delta (20).

A study by Szmyd et al. found a significant discrepancy between students' willingness to get vaccinated and their year of study. On the contrary, we found no correlation between vaccination status and the year of study. This may be due to the fact that at the time of our study, vaccines were already widely available to medical students and public vaccination campaigns were made by national and local governments.

### Role of universities regarding education in times of pandemic

As reported by Barello et al. in most universities around the world, education about virology has been omitted partially or totally (21). Others even suggested that vaccinology remained a “hobby” in some pediatric postgraduate studies (22).

In our survey, we evaluated the state of knowledge of students regarding SARS-CoV-2 by asking to point out the correct ways of transmission of the virus. It turned out that only 27.09% answered correctly all the questions. We found no correlation between the level of knowledge of students and their vaccination status. In 2017, a survey study among Polish students found that “vaccination knowledge scores of vaccination opponents turned out to be significantly lower than in vaccination proponents” (23). Our results may be explained by the one-of-a-kind status of the SARS-CoV-2 virus as a pandemic that affected the lives of most people worldwide and made even vaccination opponents more willing to get informed.

Most students felt well informed by their university about the COVID-19 symptoms (75.5% in ED and 77.6% in PD). However, students felt less informed by their university about the COVID-19 consequences (64.5% in ED and 64% in PD). This pandemic has been an educational challenge for teaching entities who had to find new ways to communicate with their students while taking into account the very dynamic character of the disease and the evolution of research. A study in 2017 in France found that despite lectures being the main type of educational tool, they were found to be effective by only 11% of the students (24). They also found that practical training was associated with better results. The nature of COVID-19 rendered most medical education online (25). Our findings reveal that short videos are the preferred tool for most students, followed by articles, and lectures are the second least preferred method of education. We hypothesized that students at universities that provided educational tools about COVID-19 were more prone to be vaccinated. Our results showed that such a relationship existed but only to a small extent (96.08% are vaccinated at universities with good educational tools vs. 91.46% at universities without). This might be explained by the fact that students also found information outside their university curriculum, for example, on social media. We believe that our results deserve the interest of Polish higher education authorities, as they underline the importance of adapting to new means of communication with students to enhance knowledge and communication with their university. Our results showed that among unvaccinated students, 35.21% of the students did not feel well informed by their university about COVID-19 prevention, while among vaccinated students the ratio was 17.10%.

### Limitations and strengths of the study

We recognize some limitations to our work. By nature, self-reported studies carry some forms of bias. Even if the link to our survey was sent only to students at healthcare universities, we cannot be sure that only the target of our survey responded to it. Additionally, the nature of the COVID-19 pandemic presents a bias that is hard to quantify as students are more likely to get vaccinated and seek information about the virus. A study found a similar bias when investigating the adherence to influenza vaccination during the H1N1 pandemic (26). Moreover, the method applied in this research was created by the authors to obtain specific statistical results reflecting features of the investigated phenomenon, therefore it was not validated psychometrically. This kind of validation, as well as an update of the questions asked, would be performed in case the research continues in the future.

The main strengths of our study are as follows. To the best of our knowledge, this is the first study to compare ED and PD in Poland, assessing the students' knowledge about COVID-19, preferred ways of education, usage of personal protective equipment, and the presence of a clear system of monitoring infections at different universities. We had a large sample from different fields of study, such as medicine, dentistry, public health, and pharmacy, from all parts of Poland. Our group from the English division was composed mostly of students from the medical department, in contrast to the Polish division students, who represented all the fields of healthcare sciences. This can be explained by the fact that at most Polish universities, only medical studies are available in English. Therefore, our English group cannot give a full perspective on the attitudes of students from other healthcare fields. Vaccines were already widely available to the public at the time of our study. Additionally, our survey was conducted a few days after the appearance of the Omicron variant and is relevant to public policymakers. We find it crucial to understand medical students' opinions about their education on current issues such as the SARS-CoV-2. Even though the pandemic will not last forever, the obtained knowledge about the university's role or individual interests' role can be applied in many different life situations. Although universities could not exist without their students, sometimes it gets omitted to understand their expectations or fears.

## Data availability statement

The raw data supporting the conclusions of this article will be made available by the authors, without undue reservation.

## Ethics statement

The studies involving human participants were reviewed and approved by Bioethical Committee of Wroclaw Medical University. The patients/participants provided their written informed consent to participate in this study.

## Author contributions

GS and AJ wrote the text. BK reviewed the text. MP did the statistical calculations. AS helped with designing the survey. AJ, GS, AS, and MP participated in the review process. AS and MP organized the funding. All authors contributed to the article and approved the submitted version.

## Funding

All the calculations and analyses were performed in cooperation with data scientists from the consortium HeartBIT_4.0—Application of Innovative Medical Data Science Technologies for Heart Diseases. HeartBIT_4.0 project has received funding from the European Union's Horizon 2020 research and innovation program under grant agreement number 857446.

## Conflict of interest

The authors declare that the research was conducted in the absence of any commercial or financial relationships that could be construed as a potential conflict of interest.

## Publisher's note

All claims expressed in this article are solely those of the authors and do not necessarily represent those of their affiliated organizations, or those of the publisher, the editors and the reviewers. Any product that may be evaluated in this article, or claim that may be made by its manufacturer, is not guaranteed or endorsed by the publisher.
